# Comparison of postoperative visual performance between bifocal and trifocal intraocular Lens based on randomized controlled trails: a meta-analysis

**DOI:** 10.1186/s12886-019-1078-1

**Published:** 2019-03-14

**Authors:** Shanshan Jin, David S. Friedman, Kai Cao, Mayinuer Yusufu, Jingshang Zhang, Jinda Wang, Simeng Hou, Guyu Zhu, Bingsong Wang, Ying Xiong, Jing Li, Xiaoxia Li, Hailong He, Xiuhua Wan

**Affiliations:** 1 Beijing Institute of Ophthalmology, Beijing Tongren Eye CenterBeijing Tongren Hospital of Capital Medical University, Beijing Key Laboratory of Ophthalmology and Visual Sciences, Beijing, China; 2 Dana Center for Preventive Ophthalmology, The Wilmer Eye Institute, Johns Hopkins University School of Medicine, Baltimore, MD USA

**Keywords:** Bifocal, Trifocal, Intraocular lenses, Cataract surgery, Randomized, IOLs, Meta-analysis

## Abstract

**Background:**

To compare the clinical performance of bifocal and trifocal intraocular lenses (IOLs) in cataract surgery, a meta-analysis on randomized controlled trials was conducted.

**Methods:**

A comprehensive literature retrieval of PubMed, Science Direct and EMBASE was performed in this systematic review. Clinical outcomes included visual acuity (VA), contrast sensitivity (CS), spectacle independence, postoperative refraction and surgical satisfaction.

**Results:**

There were 8 RCTs included in this study. The difference of uncorrected near VA (UNVA) between the bifocal IOLs and trifocal IOLs had no significance [MD = 0.02, 95%CI: (− 0.03,0.06)]. There was no significant difference in the distant-corrected near VA (DCNVA) with MD of 0.04 [95%CI (− 0.02, 0.10)]. Compared with trifocal group, the uncorrected intermediate visual acuity (UIVA) [MD = 0.09,95%CI:(0.01,0.17)] was significantly worse in the bifocal group. No difference was found in distance-corrected intermediate VA (DCIVA) [MD = 0.09, 95%CI: (− 0.04, 0.23)] between two groups. Analysis on AT LISA subgroup indicated the bifocal group had worse intermediate VA than trifocal group (AT LISA tri 839 M) [MD = 0.18, 95%CI: (0.12, 0.24) for UIVA and MD = 0.19, 95%CI: (0.13, 0.25) for DCIVA]. However, there was no statistically significant difference between the two groups in the uncorrected distance VA (UDVA) and corrected distance visual acuity (CDVA) [MD = 0.01, 95%CI: (− 0.01,0.04) for UDVA; MD = 0.00, 95%CI: (− 0.01,0.01) for CDVA].

The postoperative refraction of bifocal group was similar to that of trifocal group [MD = -0.08, 95% CI: (− 0.19, 0.03) for spherical equivalent; MD = -0.09, 95%CI: (− 0.21, 0.03) for cylinder; MD = -0.09, 95% CI: (− 0.27, 0.08) for sphere]. No difference was found for spectacle independence, posterior capsular opacification (PCO) incidence and patient satisfaction between bifocal IOLs and trifocal IOLs. [RR = 0.89, 95% CI: (0.71, 1.12) for spectacle independence; RR = 1.81, 95% CI: (0.50, 6.54) for PCO incidence; RR = 0.98, 5% CI: (0.86, 1.12) for patient satisfaction].

**Conclusion:**

Patients receiving trifocal IOLs, especially AT LISA tri 839 M, have a better intermediate VA than those receiving bifocal IOLs. Near and distance visual performance, spectacle independence, postoperative refraction and surgical satisfaction of bifocal IOLs were similar to those of trifocal IOLs.

**Electronic supplementary material:**

The online version of this article (10.1186/s12886-019-1078-1) contains supplementary material, which is available to authorized users.

## Background

Cataract is the clouding of the normally clear crystalline lens or loss of transparency, which reduces the amount of incoming light and impairs visual perception, and it is the leading cause of vision impairment and blindness worldwide. Data from the World Health Organization (WHO) showed that cataract accounted for approximately 50% of blindness worldwide [1]. In China, blindness and low vision affects about 5.8% of Chinese aged 50 and above [2]. Given the rapid population aging and high prevalence of age-related cataract (ARC) in China, especially in rural areas [3, 4], it is expected that an increasing number of population will suffer from cataract.

It is generally acknowledged that cataract surgery is the most cost-effective way for restoring vision. Harold Ridley performed the first cataract extraction with implantation of intraocular lens (IOLs) in London in 1949. Nowadays, cataract surgery has been modified into a highly specialized procedure, and the IOLs technique has also been developing constantly [5]. After the monofocal IOLs implementation, other IOLs such as multifocal IOLs (diffractive, refractive) and accommodating type of IOLs, were successively devised to correct not only cloudy lens but also astigmatism and presbyopia. Nowadays, the most commonly used multifocal IOLs in clinical practice are bifocal and trifocal IOLs. Therefore, this systematic review aims to compare the clinical visual performance of bifocal and trifocal IOLs, thereby providing solid evidence for better clinical practice.

## Methods

### Search strategy and inclusion criteria

Inclusion and exclusion criteria: 1. The study subjects should be patients with age-related cataract (P) who received cataract extraction with bifocal (I) or trifocal intraocular lens (C) implantation. The visual performance was evaluated as visual acuity (VA) including uncorrected, corrected and distance-corrected (near, intermediate and distant) performance, refraction cylinder, spherical equivalent refraction, spectacle independence, and patient satisfaction after cataract surgery (O); 2. Only randomized controlled clinical trials were included; 3. Studies without detailed outcome of postoperative visual performance of patient were excluded; 4. Non-English publications were excluded.

**Fig. 1:**
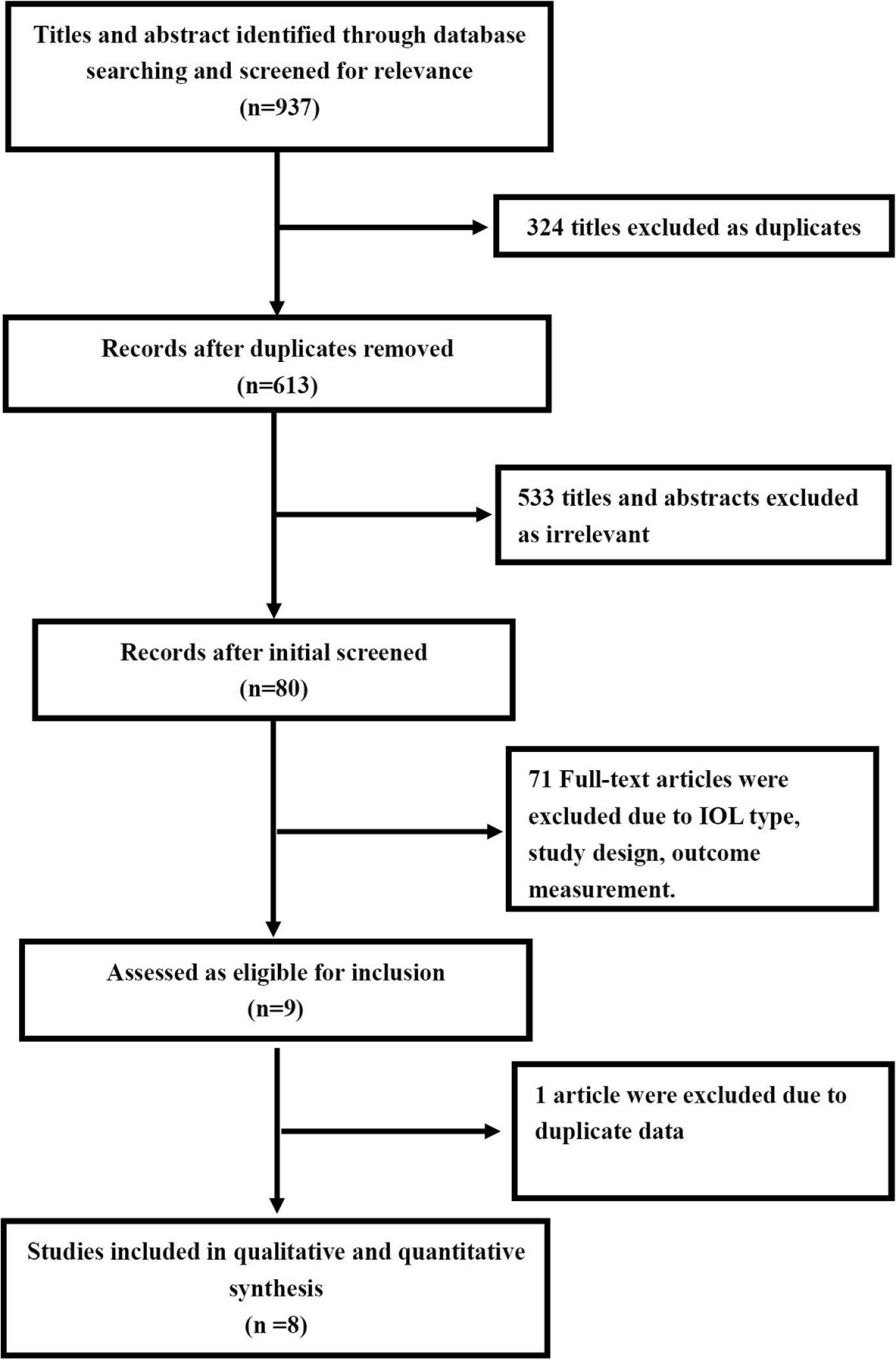
Flow chart of paper selection

Search strategy: A comprehensive search strategy for PubMed, Science direct and EMBASE was conducted. Literature published between 2007 and October 2017 was included. One or a combination of the following terms was used in the search: intraocular lenses, IOL, bifocal, trifocal, cataract surgery, comparison, random. Details of the search strategy are available in Appendix [see Additional file 1]. Two investigators (Shanshan Jin and Kai Cao) screened the articles independently. In case of disagreements, the third investigator (Xiuhua Wan) would engage in the discussion to reach consensus. Figure 1 shows the study selection process.

### Data extraction

Data were independently extracted from each study by two investigators using pre-defined data fields. Data on visual acuity reported as log MAR VA were extracted as the primary outcome. The data on near VA and intermediate VA at distance of 40 cm and 66 cm would be extracted if the VA was measured at more than one distance. Data on spectacle independence, spherical equivalent refraction refractive cylinder and surgical complications were collected as the secondary outcomes. For studies with more than one postoperative follow-up interval, the data on the postoperative visual performance at last follow-up visit were extracted. Mean ± SD was extracted for continuous data, and for categorical data, the proportion of patients reporting surgical complications and spectacle independence were extracted and recorded as a percentage. In studies involving multiple groups, the sample size of control group was divided by the number of intervention groups when extracting continuous data. As to categorical data, we added up the sample size and event number of all groups, as recommended by the Cochrane handbook [6]. In case of inconsistencies, a third investigator would review the extracted results and engage in discussion until a consensus was reached.

### Quality assessment

The quality of the included RCTs was assessed using the Cochrane Risk of Bias Tool [6]. The risk of bias was accessed from the following 6 aspects: sequence generation, allocation concealment, blinding of participants and personnel, blinding of outcome assessors, incomplete outcome data, selective outcome reporting.

### Data synthesis and statistical analysis

The analysis was performed using the statistical software open source R program (Version 3.41). The mean difference (MD = mean of bifocal IOL – mean of trifocal IOL) with a 95% confidence interval (95% CI) was adopted for continuous outcomes. If “0” falls into the confidence interval, the outcome would be considered not statistically significant. Categorical outcomes were estimated by RR with 95% CI. If “1” falls into the confidence interval, the outcome would be considered not statistically significant;

Heterogeneity across studies was tested with Q test and I^2^ statistic. The fixed-effect model (Mantel–Haenszel) would be used if there was no heterogeneity across these RCTs(I^2^<50%). If the *P*-value was below 0.1 and I^2^ was above 50%, the heterogeneity across these studies would be considered significant. We would explore the probable reasons by reviewing the studies included. With the help of sensitivity analysis, we can detect whether the heterogeneity would decrease following exclusion of each study one by one. If not, a subgroup analysis would be performed according to the clinical characteristics of these studies. If the heterogeneity did not decrease in sensitivity analysis and subgroup analysis, the DerSimonian and Laird random-effects model would be adopted to calculate pooled effect size [7, 8].

## Result

### Characteristics of included studies

Table 1describes the characteristics of the 8 included studies [9–16]. The median follow-up time was 18 months (Range: 3–24), and most subjects were over 60 years old. The included studies were published from 2014 to 2017.

**Table 1:** Characteristics of the included RCT studies (*n* = 8)

Study	Year	Site	Designs	Bifocal			Trifocal			Follow up (mouth)
				Age (Mean ± SD, year)	N	IOL types	Age (Mean ± SD, year)	N	IOL types	
Gundersen KG	2016	Norway	RCT	70.2 ± 7.8	11	ReSTOR SND1T(Toric)	62.1 ± 7.5	11	FineVision Toric	3
Jonker SM	2015	The Netherlands	RCT	64.0 ± 8.8	13	ReSTOR+ 3.0D (SN6AD1)	62.6 ± 8.8	15	Finevision Micro F	6
Alio JL	2017	New Zealand	RCT	63.2 ± 7.7	17/15	AT LISA 809 M/ReSTOR (SN6AD1)	63.2 ± 7.7	17	AT LISA tri 839MP	6
Bilbao-Calabuig R	2016	Spain	RCT	56.3 ± 6.9	11	ReSTOR (SN6AD2/SN6AD1)	56.3 ± 6.9	12	FineVision	3
Cochener B	2016	France	RCT	60.6 ± 9.1	12	Tecnis ZMB00	58.7 ± 6.4	15	FineVision Micro F	6
Mojzis P	2014	Czech	RCT	62.3 ± 5.7	15	AT LISA 801	55.2 ± 7.0	15	AT LISA tri 839MP	3
Mojzis P	2017	Czech	RCT	NR	18	AT LISA 801	NR	20	AT LISA tri 839MP	12
Gundersen KG	2016	Norway	RCT	53 ± 8	30	ReSTOR (SN6AD1/ SN6AD2)	65 ± 9	25	AT LISA tri839MP	24

*N* The number of people received the cataract surgery

*NR* Not report

### Risk of bias assessment

**Fig. 2:**
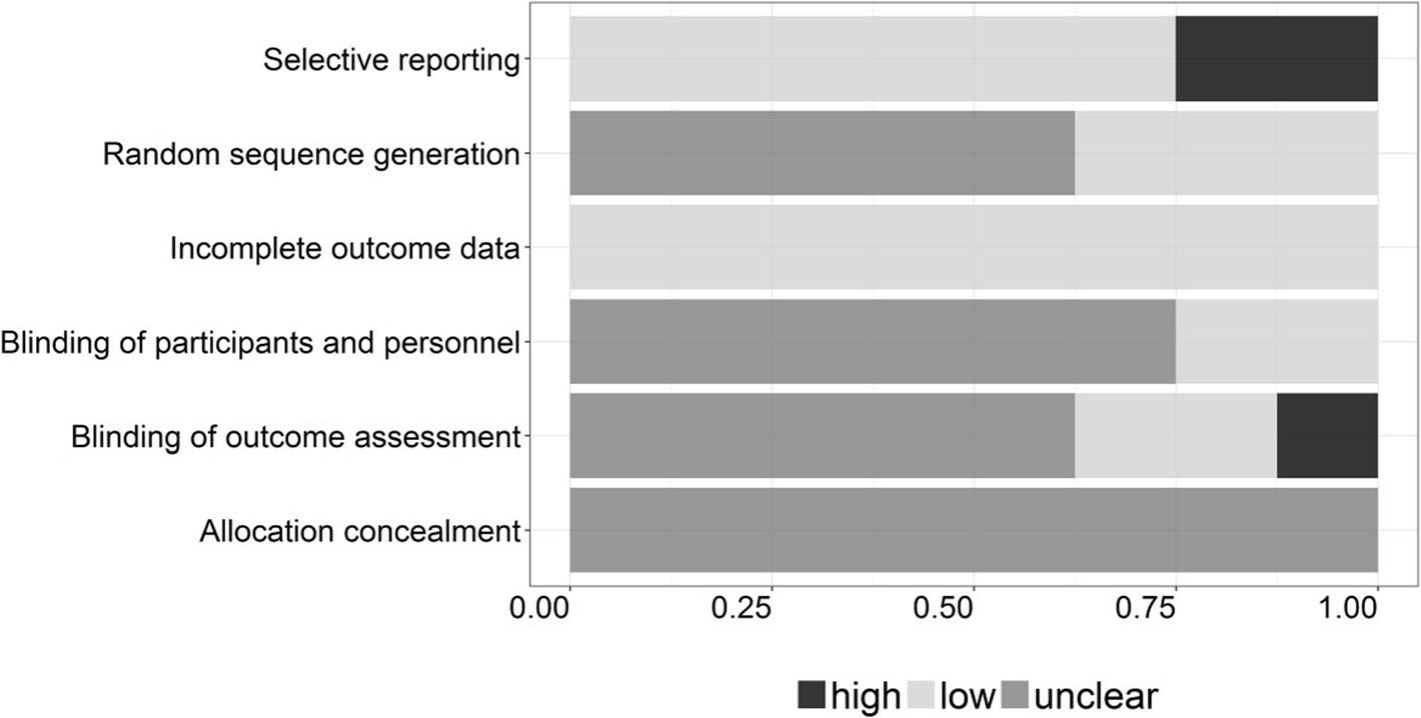
“Risk of bias” graph: Each risk of bias item presented as percentages across all included studies

**Fig. 3:**
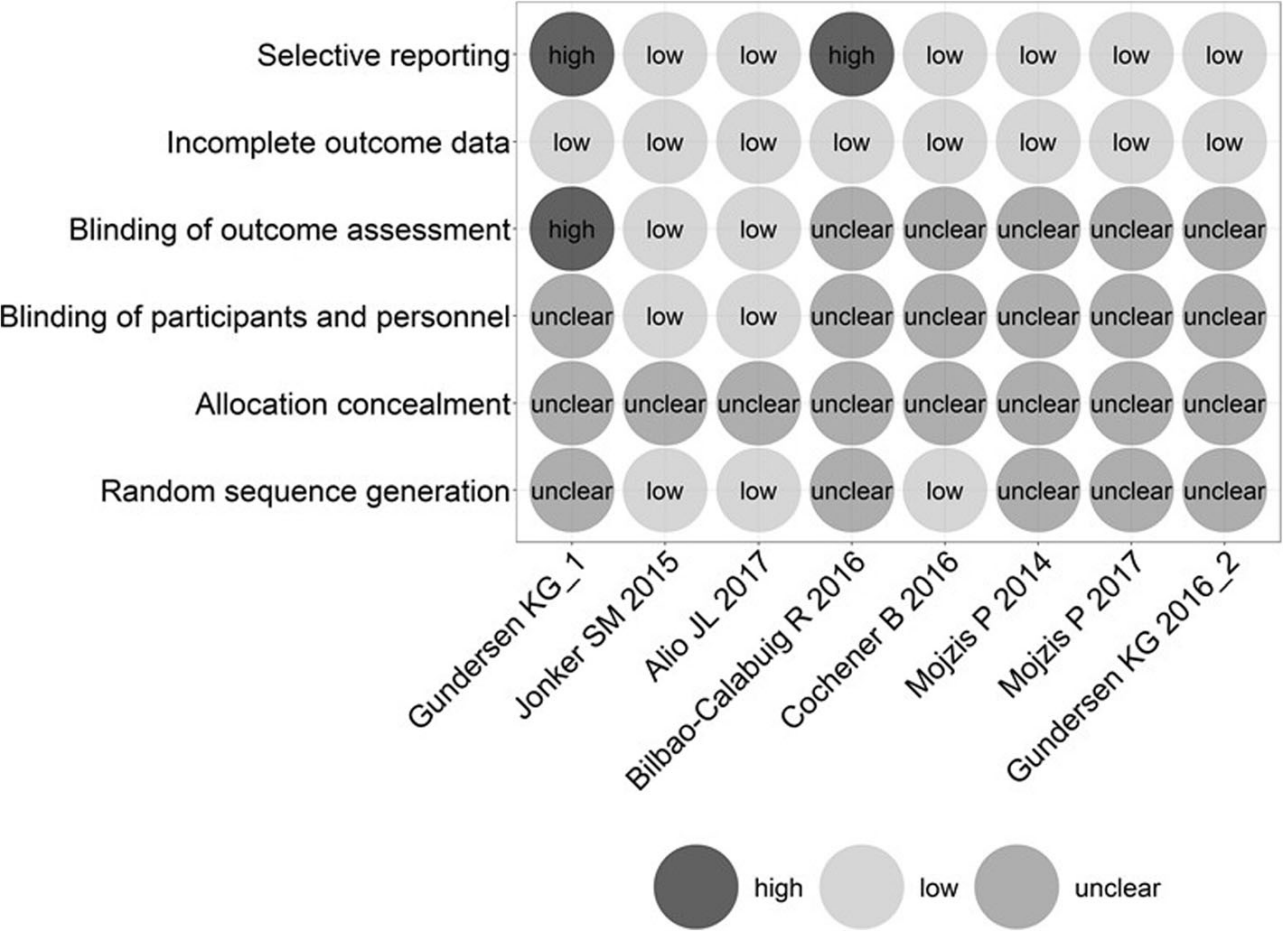
“Risk of bias” summary: Each risk of bias item for each included study

Three studies [11, 14, 15] described the method for generating random sequence, and 2 of them [11, 14] applied the blind method. Gundersen KG [12] reported that the outcome examiner was not masked to the lens type in his study. The details of risk assessment for each item of these RCTs were shown in the Figs. 2 and 3.

### Near visual acuity

**Fig. 4:**
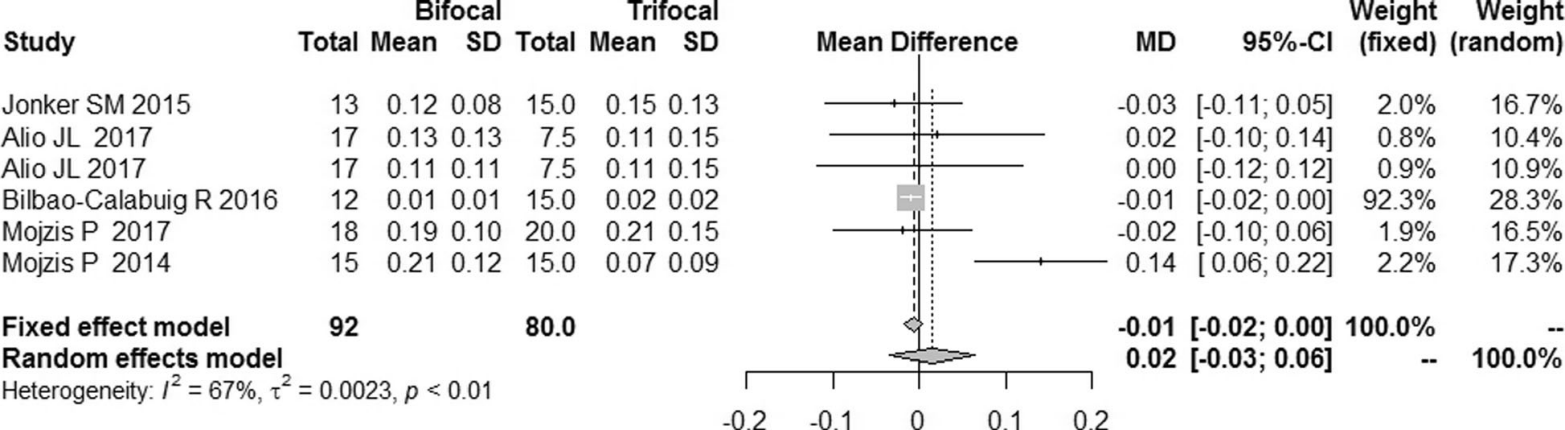
Forest plot of UNVA

**Fig. 5:**
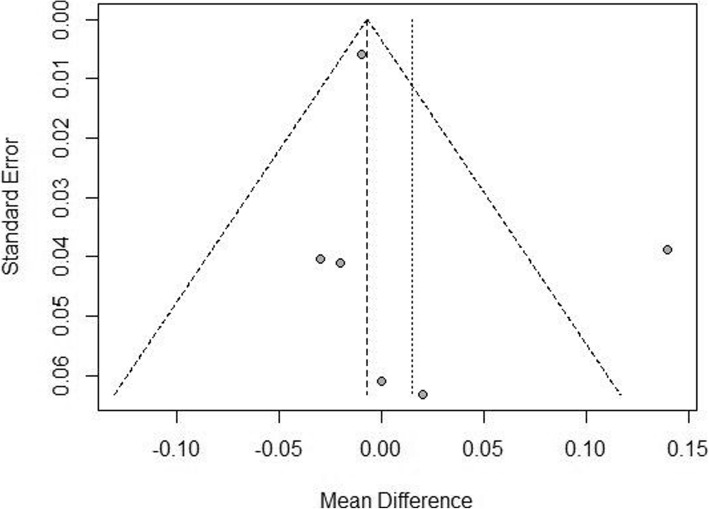
Funnel plot of UNVA

**Fig. 6:**
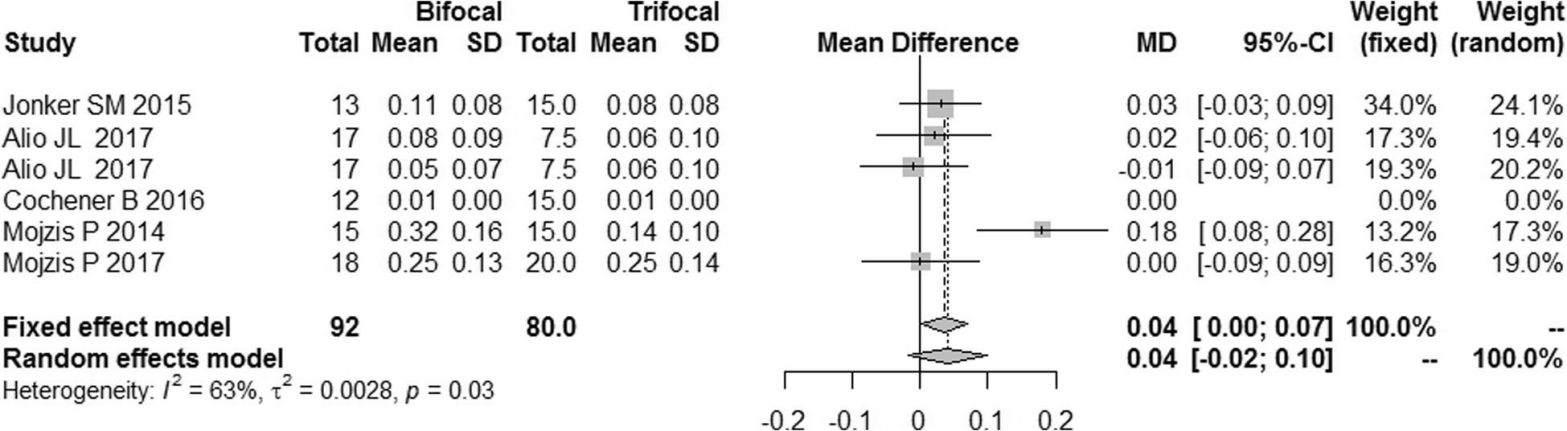
Forest plot of DCNVA

There were 5 studies reporting uncorrected near visual acuity (UNVA) and distant-corrected near visual acuity (DCNVA), and the I^2^ was 67 and 63% respectively. Sensitivity analyses revealed that the study published in 2014 by Mojzis P et al. [9] was the source of heterogeneity for the UNVA and DCNVA. After the study by Mojzis P et al. was excluded, the I^2^ were reduced to 0% for both UNVA and DCNVA. The difference of UNVA between the bifocal IOLs and trifocal IOLs had no significance [MD = 0.02, 95%CI: (− 0.03,0.06)] (Fig. 4). No publication bias was found (Fig. 5). The DCNVA of bifocal IOLs was similar to that of trifocal IOLs [MD = 0.04, 95%CI: (− 0.02,0.10) for DCNVA] (Fig. 6).

### Intermediate visual acuity

**Fig. 7:**
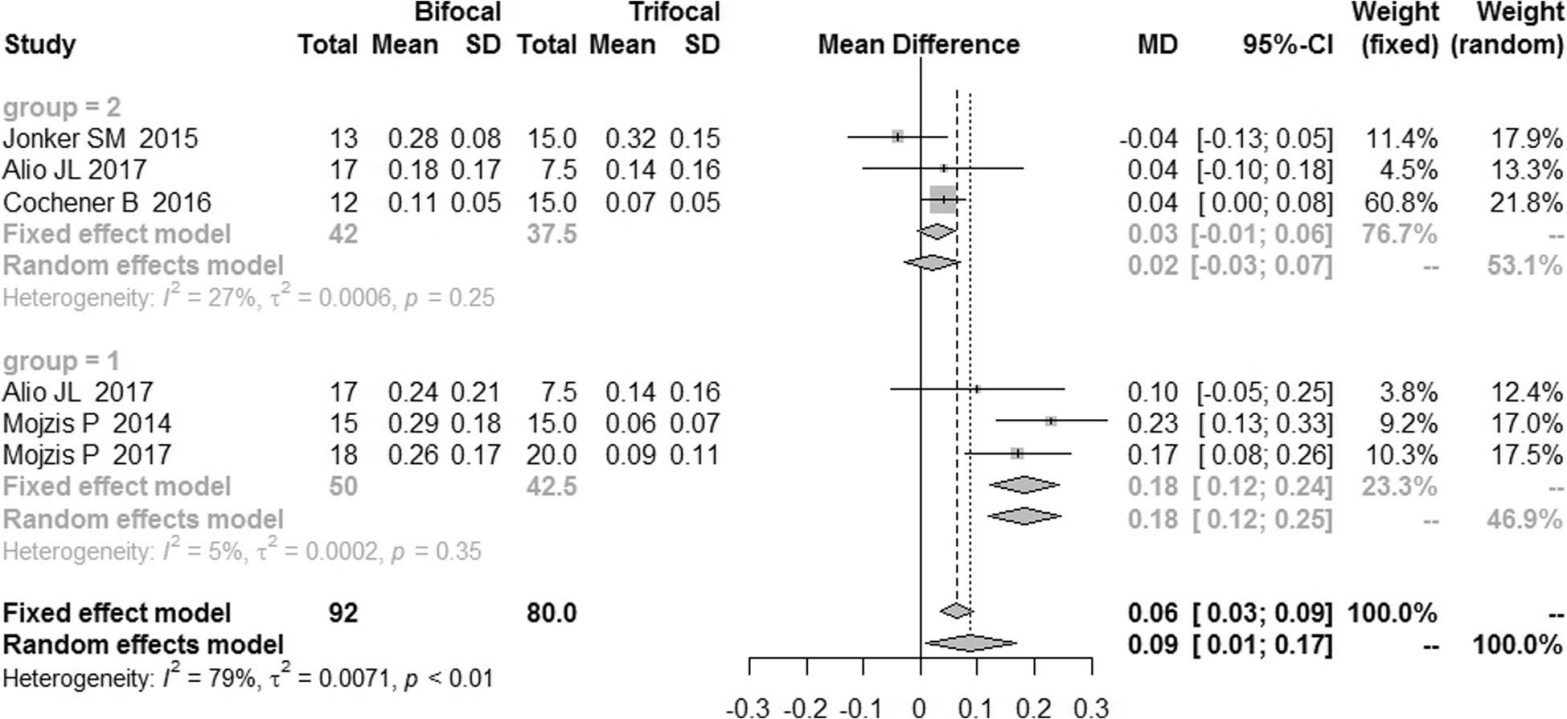
Forest plot of UIVA

**Fig. 8:**
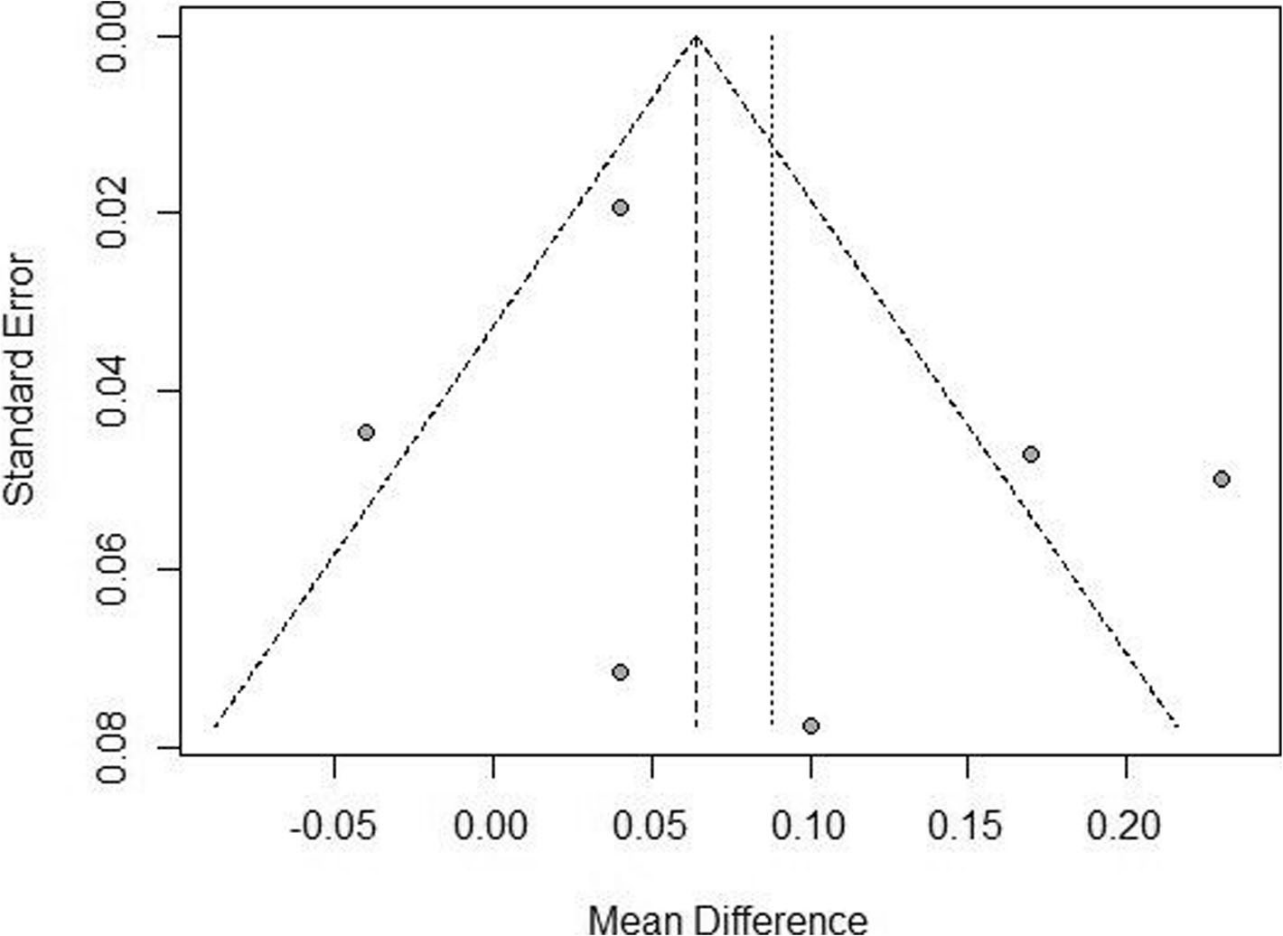
Funnel plot of UIVA

**Fig. 9:**
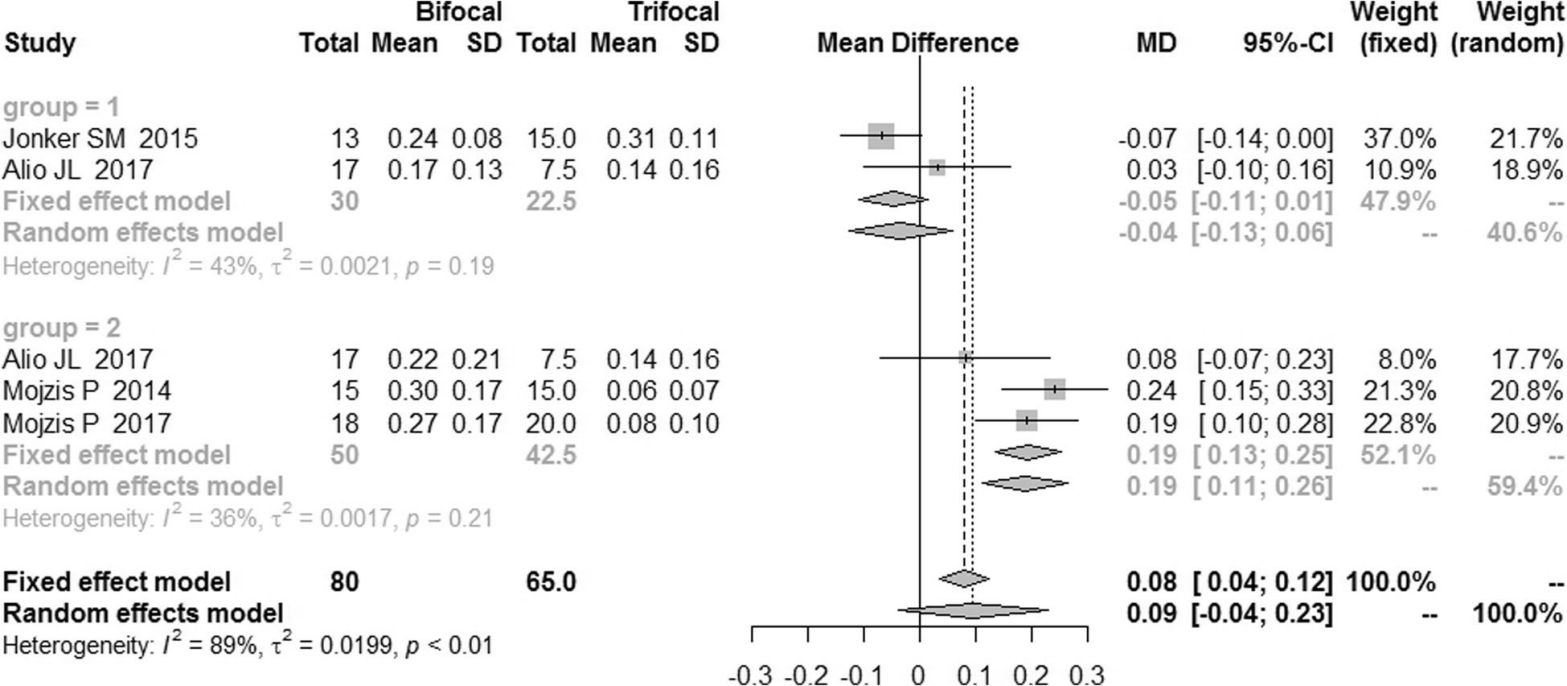
Forest plot of DCIVA

There was a high heterogeneity of uncorrected intermediate visual acuity (UIVA) and distance-corrected intermediate visual acuity (DCIVA), which was 79 and 89% respectively. Therefore, the random effects model was applied. As shown in Fig. 7, the bifocal IOLs had a significantly worse performance in UIVA compared with trifocal IOLs with a MD of 0.09 [95%CI: (0.01,0.17)]. No publication bias was revealed (Fig. 8), and there was no difference in DCIVA [MD = 0.09, 95%CI: (− 0.04, 0.23)] (Fig. 9).

The sensitivity analysis failed to reveal the source of heterogeneity, thus the subgroup analysis was conducted, which reduced the I^2^ to 5 and 36% respectively as shown in Figs. 7 and 9. These RCTs were grouped by the type of IOLs. In the AT LISA subgroup, UIVA and DCIVA of the bifocal IOLs were significantly worse than those of trifocal IOLs [MD = 0.18, 95%CI: (0.12, 0.24) for UIVA and MD = 0.19, 95%CI: (0.13, 0.25) for DCIVA].

**Fig. 10:**
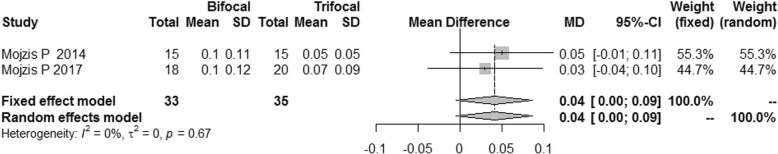
Forest plot of CIVA

As shown in Fig. 10, there were 2 RCTs reporting the outcomes of corrected intermediate visual acuity (CIVA). Although Mojzis P’s study published in 2014 [9] found statistical difference between bifocal and trifocal IOLs, our study found no difference between bifocal and trifocal IOLs [MD = 0.04, 95% CI: (0.00, 0.09)]. No publication bias was revealed, and the outcome of the Egger’s test for each index was shown in Table 2.

**Table 2:** Egger’s test for each outcome

Indicators	t	df	p
UNVA	0.877	4	0.430
DCNVA	0.741	3	0.513
UIVA	0.814	4	0.461
DCIVA	0.543	3	0.625
UDVA	−1.079	3	0.360
CDVA	0.101	5	0.923
Spherical equivalent refraction	−0.403	4	0.707
Refractive cylinder	−0.073	3	0.946
Residual sphere	0.316	1	0.805
Patients satisfaction	−0.177	1	0.888

### Distant visual acuity

**Fig. 11:**
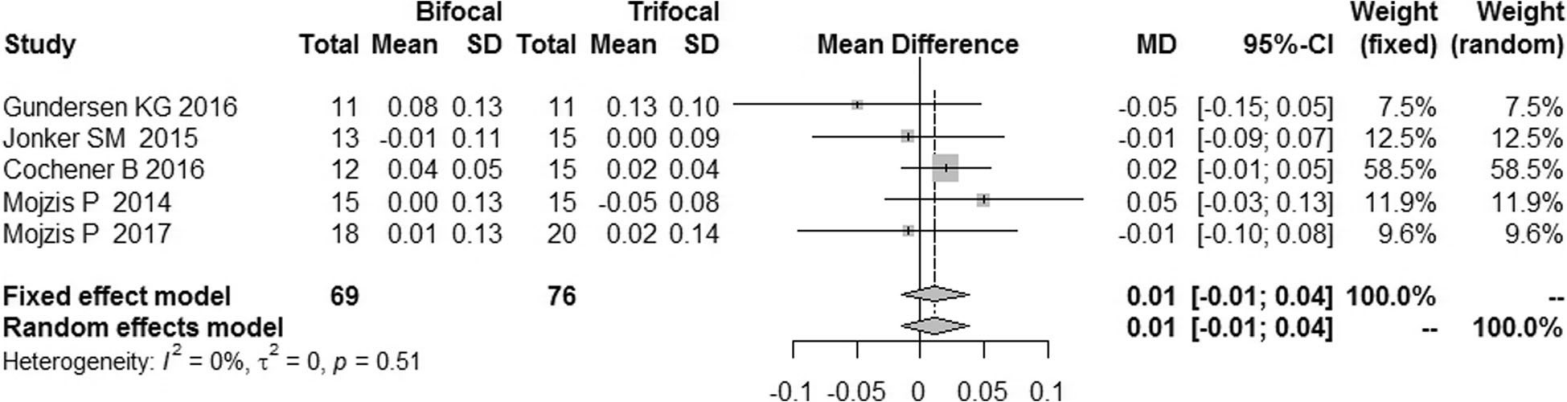
Forest plot of UDVA

**Fig. 12:**
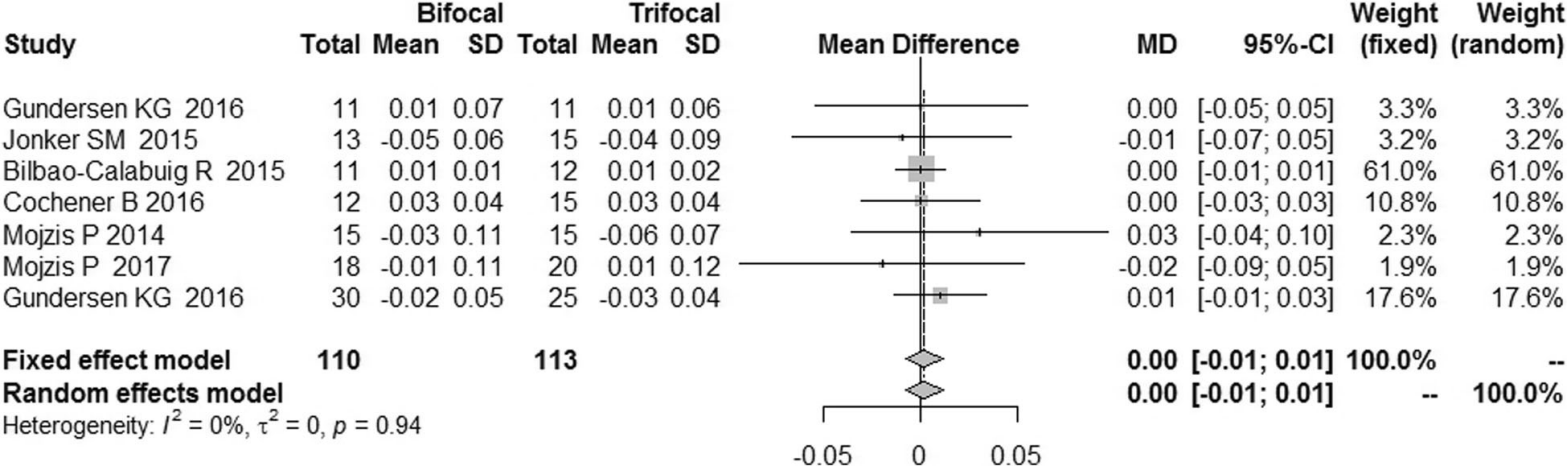
Forest plot of CDVA

There was no statistical heterogeneity among these RCTs (I^2^ = 0%) in terms of uncorrected distance visual acuity (UDVA) and corrected distance visual acuity (CDVA). We found no statistically significant difference between the two groups, and the distant VA results were similar [MD = 0.01, 95%CI: (− 0.01,0.04) for UDVA; MD = 0.00, 95%CI: (− 0.01,0.01) for CDVA] (Figs.11 and 12).

### Contrast sensitivity

Contrast sensitivity (CS) was reported in 6 studies [9–11, 14–16]. These studies indicated the highest CS was in 6 Cycles per Degree. Only Jonker ‘s study [11] found the mean mesopic CS was higher in the bifocal groups, while the rest revealed no difference between the two groups. Mojzis P’s studies published in 2014 and 2017 [9, 14] indicated the CS of trifocal groups was higher than that of bifocals under 3 Cycles per Degree.

### Spectacle independence

**Fig. 13:**
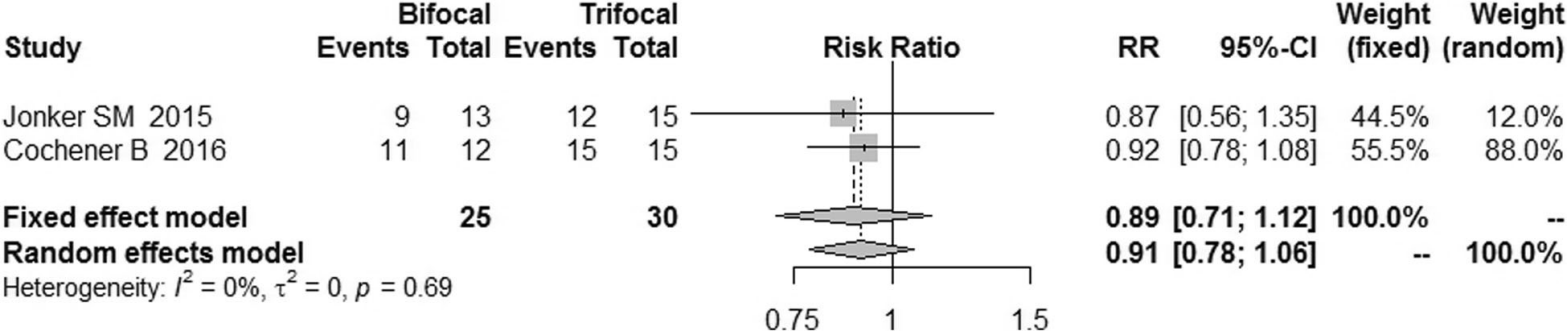
Forest plot of spectacle independence

Only two studies reported data on spectacle independence (Fig. 13). The spectacle independence of trifocal group was similar to that of the bifocal group, with a RR of 0.89 [95% CI: (0.71, 1.12)].

### Postoperative refraction

**Fig. 14:**
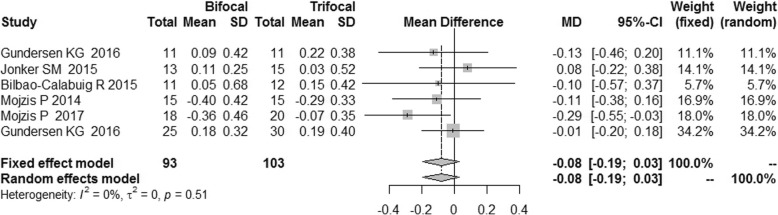
Forest plot of spherical equivalent refraction

**Fig. 15:**
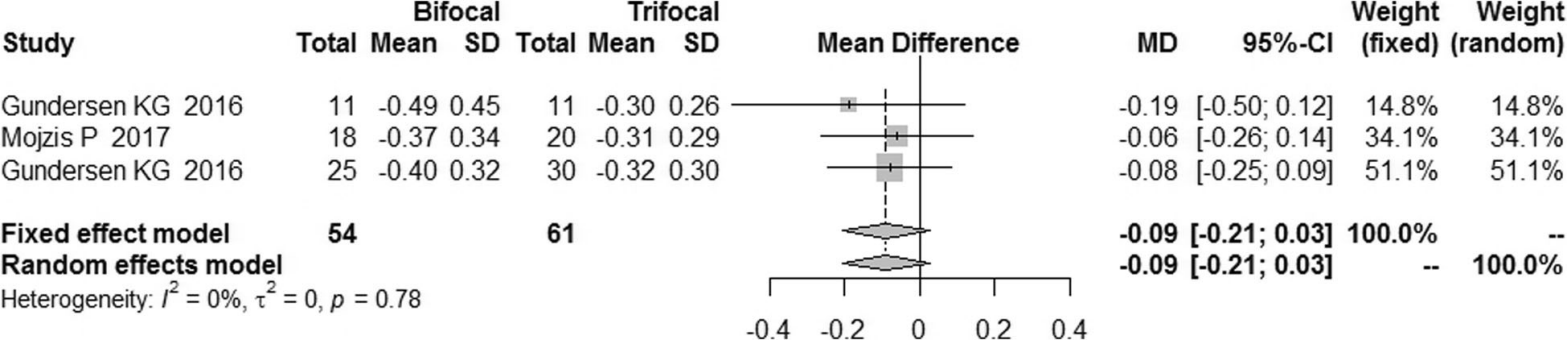
Forest plot of refractive cylinder

**Fig. 16:**
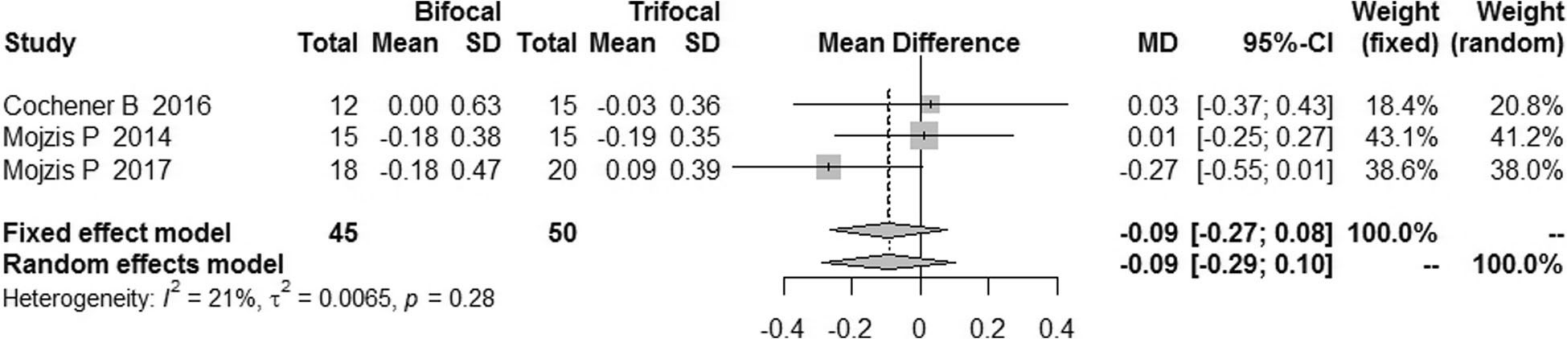
Forest plot of residual sphere

We did not find statistically significant difference in the postoperative refraction between the bifocal and trifocal groups [MD = -0.08, 95% CI: (− 0.19, 0.03) for spherical equivalent; MD = -0.09, 95%CI: (− 0.21, 0.03) for cylinder; MD = -0.09, 95% CI: (− 0.27, 0.08) for sphere]. I^2^ of these indexes was 0 and 21% respectively (Figs. 14, 15 and 16).

### Posterior capsular opacification (PCO)

**Fig. 17:**
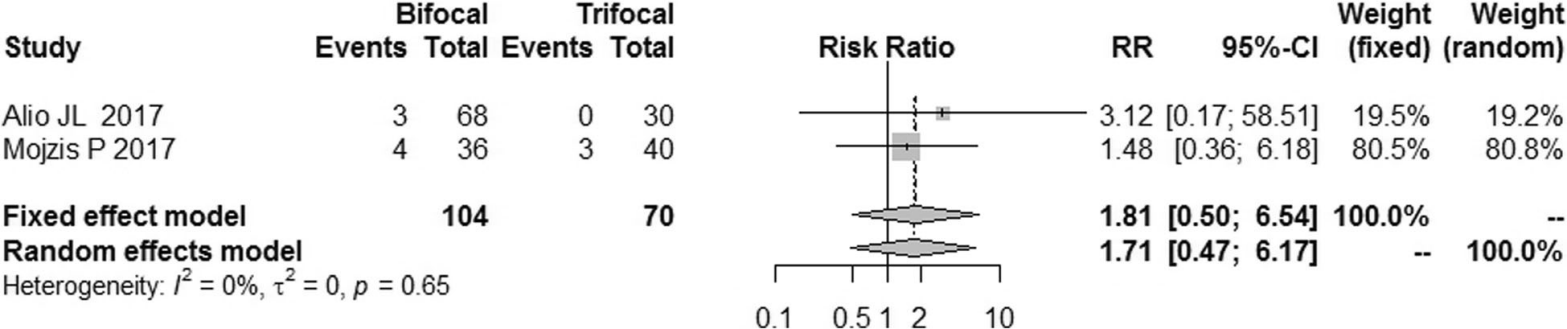
Forest plot of PCO

As shown in Fig. 17, the PCO incidence in the bifocal group was similar to that in trifocal group, with a RR of 1.81, [95% CI: (0.50, 6.54)]. Although there were only two studies reporting the data on PCO, the outcomes of these two studies (I^2^ = 0%) showed a high level of consistency.

### Surgical satisfaction

**Fig. 18:**
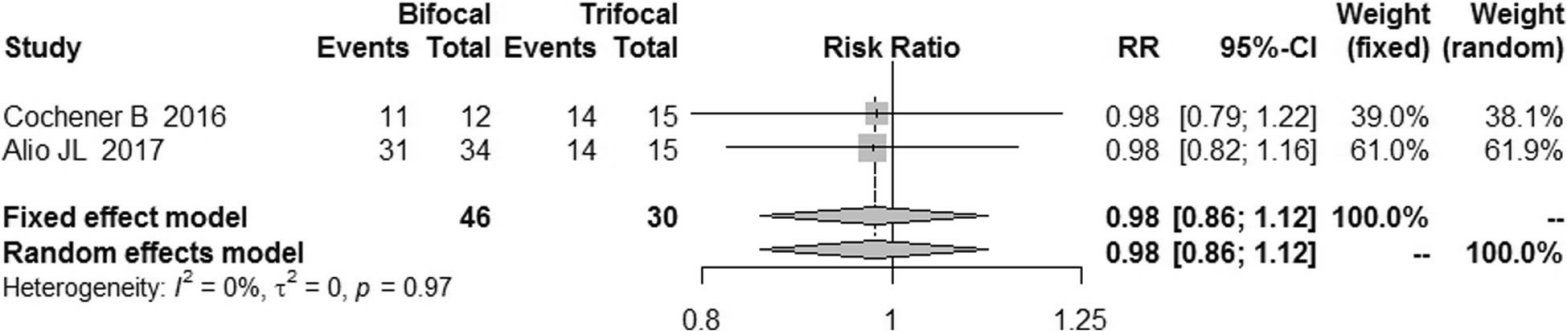
Forest plot of patient satisfaction

Three studies [11, 15, 16] reported the data on patient satisfaction and all of them recorded a high level of patient satisfaction after the surgery in both bifocal and trifocal group, with a high level of consistency (I^2^ = 0%). Our study found no difference for surgical satisfaction between bifocal and trifocal groups [RR = 0.98, 95% CI: (0.86, 1.12)] (Fig. 18).

## Discussion

Admittedly, the level of evidence of RCT study is the highest according to the categories and recommendations of medical studies in the field of Evidence-based medicine (EBM) [17]_._ This study is the systematic review on RCTs comparing the visual performance of bifocal and trifocal IOLs. As the results showed, we found there was a statistically significant difference in intermediate visual acuity between the two groups. However, no statistical significance was found in other variables in our study.

In this systematic review, two studies [11, 16] reported the process of blinding. In fact, it is difficult to mask the patients and the surgeons about the type of IOLs implanted in the cataract surgery. Moreover, since no study reported the process of allocation concealment, it is possible that these studies had selection bias. Meanwhile two studies [10, 12] reported reporting bias. Consequently, the pooled effect of this meta-analysis might be affected by these biases.

As indicated by the sensitivity analyses, the study of Mojzis P et al. was the source of heterogeneity of UNVA and DCNVA. The possible explanation might be that the follow-up interval of this study was 3 months while the interval of other studies was 6 mouths. In this study, no statistically significant difference between the two types of IOLs was found for UNVA and DCNVA. There are two possible reasons: 1. despite the inconsistent outcomes of these RCTs, most of them reported no statistical differencein the near vision improvement; 2. multifocal IOLs designs were initially bifocal and were used to improve the postoperative vision acuity and reduce spectacle dependence at near distance. In fact, with the evolution of multifocal IOLs, both bifocal and trifocal IOLs showed excellent near visual performance [18, 19].

The intermediate vision greatly affects our daily work, exercise and social life, especially the computer uses in daily office work. Hence, it is also an important factor for the postoperative satisfaction of cataract patients [20, 21]. Our study revealed a better intermediate vision in the trifocal IOL implantation group, which makes sense given the trifocal IOLs was originally devised to overcome the limitation regarding the postoperative intermediate visual function. Since the outcomes of UIVA and DCIVA might be inconvincible due to the heterogeneity (I^2^>79%), a subgroup analysis on different types of the bifocal IOLs and follow-up time was conducted. The heterogeneity of these RCTs was eliminated in the subgroup analysis on bifocal IOLs types, but not follow-up time. In the AT LISA subgroup, UIVA and DCIVA were significantly better in the trifocal IOLs group (AT LISA tri 839 M). Many researches have confirmed better intermediate visual performance of AT LISA tri 839 M trifocal IOLs compared with the AT LISA bifocal IOLS with both clinical study and the optical bench [22–25] that proved the AT LISA tri 839 M can provide a third effective focus. Additionally, no difference was found for the UIVA and DCIVA between the bifocal (Re STOR bifocal IOLs) and the trifocal IOLs (Fine Vision trifocal IOLs) in the other subgroup. Plaza-Puche AB [26] and Ruiz-Alcocer J [27] have demonstrated that the AT LISA tri IOLs supports better intermediate visual outcome in comparison with Fine Vision trifocal IOLs in both clinical experiment and optic blench. Furthermore, Plaza-Puche AB corroborated that there was no difference in intermediate vision between the Fine Vision IOLs and the AcrySof ReSTOR bifocal IOLs, which is consistent with our results [11, 15].

Due to the lack of measurement data, no conclusive results could be drawn for contrast sensitivity. In this systematic review, studies that reported the CS suggested no difference of CS was found between bifocal and trifocal IOLs, which is in accordance with previous researches [26, 28]. Therefore, the addition of a third focal point does not seem to decrease the postoperative optical quality.

Spectacle independence is commonly used for the evaluation of the satisfaction, and life quality of cataract patients in scales like the National Eye Institute Refractive Error Correction Quality of Life Instrument-42 (NEI-RQL 42) questionnaire [29]_,_ Visual Function Index-14 (VF-14) [30]. In our study, the spectacle independence and postoperative satisfaction did not differ between the two IOLs. The lack of statistically significant difference may be attributed to the fact that both IOLs showed excellence performance of spectacle independence, which is in line with the results of previous studies [31–33]. In addition, the version of questionnaire used for spectacle independent and satisfaction in each single study was different, which may affect the result of the pooled effect.

As for the postoperative refraction, no statistically significant difference was found between the two groups. Many researches have proved that both bifocal IOLs and trifocal IOLs showed great clinical performance in terms of the refractive correction after surgery [34, 35]. Besides the postoperative refraction, there was also no difference in the PCO incidence between bifocal and trifocal groups.

Admittedly, there were some limitations in this study. First, this meta-analysis was not the first review that compares the visual performance of patients receiving bifocal or trifocal IOLs implementation [36–39]. However, this systematic review was the only one that included only RCTs, providing a higher level of evidence. Second, several studies in this meta-analysis did not report sufficient data on risk assessment, especially random sequence generation, allocation concealment and setting blinding, which may lead to bias. Third, given the number of patients in each study is relatively small, we could not draw explicit conclusion on the difference in postoperative visual performance. Last, the studies included used different types of bifocal and trifocal IOLs, thus the pooled effect might not be accurate enough.

## Conclusions

Our systematic review revealed the trifocal IOLs provide a better intermediate VA compared with bifocal IOLs, especially for the AT LISA subgroup, while the near and distant visual performance, spectacle independence, contrast sensitivity, postoperative refraction and surgical satisfaction of bifocal IOLs were similar to those of trifocal IOLs. In order to achieve more significant findings, RCTs with larger sample size should be conducted.

## Additional file

Additional file 1: Appendix. Details of the search strategy of this meta-analysis. (DOCX 14 kb)

## Abbreviations

CDVA: Corrected distance visual acuity
CS: Contrast sensitivity
DCIVA: Distance-corrected intermediate VA
DCNVA: Distant-corrected near VA
IOLs: Intraocular lens
NEI-RQL -42: National Eye Institute Refractive Error Correction Quality of Life Instrument-42 questionnaire
PCO: Posterior capsular opacification
UDVA: Uncorrected distance VA
UIVA: Uncorrected intermediate visual acuity
UNVA: Uncorrected near VA
VA: Visual acuity
VF-14: Visual Function Index-14
