# Supplementary material for: Comparison of postoperative visual performance between bifocal and trifocal intraocular Lens based on randomized controlled trails: a meta-analysis
Source: BMC Ophthalmol. 2019 Mar 14;19:78. doi: 10.1186/s12886-019-1078-1 (PMC6419463; doi:10.1186/s12886-019-1078-1)
Supplement: Supplementary file 1 — Appendix. Details of the search strategy of this meta-analysis. (DOCX 14 kb) [file 12886_2019_1078_MOESM1_ESM.docx]

**Appendix:**

For each search strategy item, there is another restriction: date between January 1, 1998 to December 31, 2017.

Search strategy for PubMed (for each search strategy item, there is another restriction: date between January 1, 1998 to December 31, 2017)

#1. intraocular[All Fields] AND IOLs[All Fields] AND ("cataract extraction"[MeSH Terms] OR ("cataract"[All Fields] AND "extraction"[All Fields]) OR "cataract extraction"[All Fields] OR ("cataract"[All Fields] AND "surgery"[All Fields]) OR "cataract surgery"[All Fields]) AND comparison[All Fields]

#2. *focal and random* and cataract

#3. Bifocal and Trifocal and comparison

#4. Trial and IOLs

Search strategy for Science Direct and EMBASE

#1. Lenses, Intraocular AND comparison AND cataract

#2. Lenses, Intraocular AND random* AND cataract

#3. Bifocal AND Trifocal Intraocular Lenses AND comparison

#4. Trial and IOLs
